# Obesity status is a risk factor for secondary surgery after neurolysis, direct nerve repair or nerve grafting in traumatic brachial plexus injury: a retrospective cohort study

**DOI:** 10.1186/s12893-020-00737-4

**Published:** 2020-04-15

**Authors:** Pichitchai Atthakomol, Kamilcan Oflazoglu, Kyle R. Eberlin, Jonathan Winograd, Neal C. Chen, Sang-Gil Lee

**Affiliations:** 1 Department of Orthopaedic Surgery, Massachusetts General Hospital, Boston, MA USA; 2 Department of Orthopaedics, Faculty of Medicine, Chiang Mai University, Chiang Mai, Thailand; 3 Musculoskeletal Science and Translational Research Center, Chiang Mai University, Chiang Mai, Thailand

**Keywords:** Obesity, Risk factor, Secondary surgery, Neurolysis, Nerve repair, Nerve grafting, Brachial plexus injury

## Abstract

**Background:**

The objective of the study was to investigate the association between obesity and the presence of secondary surgery following neurolysis, direct nerve repair, or nerve grafting in patients with traumatic brachial plexus injury.

**Methods:**

In this retrospective chart review spanning two Level I medical centers in a single metropolitan area, 57 patients who underwent neurolysis, direct nerve repair, or nerve grafting for brachial plexus injuries between 2002 and 2015 were identified. Risk regression analysis was used to evaluate the association between obesity status and secondary surgery.

**Results:**

After controlling for the confounding variables of age, high energy injury, associated shoulder dislocation and associated clavicle fracture using multivariate regression (risk regression), the risk ratio of secondary surgery in obese patients compared to non-obese patients was 6.99 (*P* = 0.028). The most common secondary surgery was tendon or local muscle transfer.

**Conclusions:**

There is an increased risk of secondary surgery in obese patients compared to non-obese patients of the same age and with the same severity of injury. The increased risk may be due to challenges related to powering a heavier upper extremity. A weight reduction program might be considered as part of the preoperative strategy.

## Background

Prior to the advent and growth in popularity of nerve transfers, traumatic injuries of the plexus were generally treated with direct repair, nerve grafting, or neurolysis. During exploration of a brachial plexus injury, the nerve can be grossly transected, a traction injury with a well-defined zone of injury, or a post-ganglionic neuroma in continuity with electrical evidence of physiological continuity [1–6]. The mechanism of injury, severity of injury, and intraoperative findings guide operative decision making [1, 4, 7–10].

Prior studies on neurolysis, direct nerve repair, nerve grafting or nerve transfer in traumatic brachial plexus injury have mainly focused on functional outcomes, typically using the Medical Research Council (MRC) scale. Poor outcomes (motor power < grade 3) has been reported in up to 40% of patients [2, 4, 7, 11–16]. Neurolysis, direct nerve repair or nerve grafting in the brachial plexus area might share the reinnervation to many muscle units which might reduce the MRC grade compared to nerve transfer. For those types of surgery, other measures of evaluating outcomes such as secondary surgery might provide different insights into brachial plexus surgery than traditional metrics. Secondary surgery might reflect failure of reinnervation or persistent dysfunction despite re-innervation and might encompass different aspects of outcomes that MRC grading or validated outcomes do not always capture.

Previous studies have reported that body mass index (BMI) may be correlated with the results of nerve transfer in brachial plexus injury patients [17–20]. However, the effect of BMI and neurolysis, direct nerve repair or nerve grafting in brachial plexus injuries is still controversial.

We aimed to examine the correlation between BMI and the presence of secondary surgery to improve upper extremity function after primary reconstruction with neurolysis, direct nerve repair, or nerve grafting procedures in patients with traumatic brachial plexus injury.

## Methods

### Study design

This study was approved by the local Institutional Review Board. In this retrospective chart review, the medical records of all adult patients with traumatic brachial plexus injury treated by eleven surgeons at two urban hospitals between January 2002 and December 2015 were included. International Classification of Disease 9 (ICD-9) codes within the defined timeframe were used to identify all patients with an injury of the brachial plexus and were cross matched with operative procedures using Current Procedural Terminology (CPT) codes (Additional File 1). We identified 529 patients aged over 18 years with both an ICD-9 and a CPT code for brachial plexus injury; each case was then manually reviewed. Only patients who were initially treated with neurolysis, direct nerve repair, or nerve grafting procedures were included in the study. After excluding patients without a traumatic plexus injury and those who were miscoded, a total of 57 patients were included in our study.

Secondary surgery was defined as any procedure after the first brachial plexus surgery performed in order to improve upper extremity function. The definition of BMI was the weight of the individual (kilograms) divided by the square of the height (meters). A previous study reported that BMI was correlated with mid upper arm circumference and might be representative of the weight of upper extremity [21].

The following data were manually gathered from the medical records:

- Baseline characteristics: age, sex, race, BMI of 30 kg/m^2^ or higher [22], and smoking status
- Injury characteristics: mechanism and cause of injury, and injury severity. Motor vehicle accidents, severe lacerations, and falls from a tree were categorized as high energy injuries. We excluded gunshot injuries from high energy injuries because previous studies have suggested that most gunshot injuries are nerve lesions continuity [6, 23].
- Initial pattern (C5-C6+/−C7, C8-T1, C5-T1) and associated injuries (e.g., clavicle fracture or shoulder dislocation).
- Treatment characteristics: time from injury to the first brachial plexus surgery and the surgical method)

Characteristics of secondary surgeries were also described in each patient, including indications for and type of secondary surgery, type of primary brachial plexus surgery, level of recovery after primary operation, type of secondary and (if applicable) third and fourth surgeries, and time from first brachial plexus surgery to secondary surgery.

### Statistical analysis

Categorical variables are reported as frequencies and percentages. Non-parametric continuous variables are reported as median and interquartile range (IQR). In bivariate analysis, associations between secondary surgery and categorical variables were calculated using the Fisher’s exact test; associations with continuous variables were calculated using the Mann-Whitney U test. A *P*-value of less than 0.05 was considered statistically significant.

The relationship between BMI and secondary surgery was evaluated with advance plots in Lowess line type. If a relationship was non-linear, we categorized patients with BMI of 30 kg/m^2^ or higher as “obese” [22].

The power of statistical analysis of association between the determinant and outcome was calculated via a matched case-control study. We interpreted the results if the power of the statistic was at least 0.8.

Multivariate regression was used to calculate risk ratios and confounders will be adjusted accordingly.

### Demographic data

Of the 57 patients with brachial plexus injury, 39 (69%) were treated with neurolysis, 15 (26%) with nerve grafting, and 3 (5%) with direct nerve repair. Forty were male (70%), the median age was 39 (IQR 18–53), most were Caucasian (75%), and did not smoke (83%). Most patients (56%) had a high energy injury. Traction was the most common mechanism of injury (88%). The median time-to-surgery was 6 months (IQR 2–10 months) (Table 1).

**Table 1:** Patient characteristics and factors associated with secondary surgery of neurolysis, direct repair, nerve grafting

Characteristic		Secondary surgery		***P*** Value
	All patients	Yes	No	
	(***n*** = 57)	(***n*** = 9)	(***n*** = 48)	
**Age, median (SD), y**	39 (16)	40 (18)	38 (16)	0.66
**Sex, n(%)**				0.11
Men	40 (70)	4 (44)	36 (75)	
Women	17 (30)	5 (56)	12 (25)	
**Race, n(%)**				0.74
White	43 (75)	8 (89)	35 (73)	
Black	6 (11)	1 (11)	5 (10)	
Hispatic	1 (2)	0 (0)	1 (2)	
Unknown	7 (12)	0 (0)	7 (15)	
**Smoking, n(%)**^**a**^				0.61
Yes	8 (17)	2 (25)	6 (16)	
No	38 (83)	6 (75)	32 (84)	
**Obesity, n(%)**^**b**^				0.007*
Yes	11 (25)	5 (71)	6 (16)	
No	33 (75)	2 (29)	31 (84)	
**Mechanism, n(%)**				0.64
Traction	50 (88)	9 (100)	41 (85)	
Gunshot	1 (2)	0 (0)	1 (2)	
Cut	6 (10)	0 (0)	6 (13)	
**High energy injury**^**c**^**, n(%)**				0.72
Yes	32 (56)	6 (67)	26 (54)	
No	25 (44)	3 (33)	22 (46)	
**Initial pattern, n(%)**				> 0.99
C5-C6+/−C7	25 (44)	4 (45)	21 (44)	
C8-T1	10 (17)	1 (10)	9 (19)	
C5-T1	22 (39)	4 (45)	18 (37)	
**Associated clavicle fracture, n(%)**				> 0.99
Yes	13 (23)	2 (22)	11 (23)	
No	44 (77)	7 (78)	37 (77)	
**Associated shoulder dislocation, n(%)**				> 0.99
Yes	5 (9)	1 (11)	4 (8)	
No	52 (91)	8 (89)	44 (92)	
**Surgical method, n(%)**				0.82
Neurolysis	39 (69)	6 (67)	33 (69)	
Nerve grafting	15 (26)	3 (33)	12 (25)	
Direct repair	3 (5)	0 (0)	3 (6)	
**Time to surgery, median (IQR), m**	6 (2–10)	10 (8–15)	5 (2–7)	0.05

^**a**^*n* = 46; ^**b**^*n* = 44,^c^High energy injury = MVA, severe laceration, falls from a tree^,^ *P value< 0.05

## Results

Nine out of 57 patients (16%) underwent secondary surgery after neurolysis (6 of 39 or 15%), direct nerve repair (0 of 3), or nerve grafting (3 of 15 or 20%). The statistical power to identify association between obesity and secondary surgery was 0.8. Obesity status was significantly associated with the presence of secondary surgery (*P* = 0.007). Bivariate analysis found no significant associations between other variables and the presence of secondary surgery (Table 1).

After controlling for confounders (age, high energy injury, associated shoulder dislocation and associated clavicle fracture) in multivariate regression, the risk ratio of secondary surgery was 6.99 between obese and non-obese patients (*P* = 0.028; Table 2).

**Table 2:** Association between obesity status and the presence of secondary surgery after adjusting confounder

Determinant	Risk ratio	*P* value	95% confident interval
**Obesity**	6.99	0.028 *	1.23–39.63
Age^a^	0.98	0.57	0.92–1.05
High energy injury^a^	2.84	0.34	0.33–24.78
Associated shoulder dislocation^a^	4.72	0.32	0.23–97.62
Associated clavicle fracture^a^	1.02	0.99	0.09–11.09

*P value< 0.05, ^a^confounder

Some patients did not appear for follow-up, so it was not possible to obtain their outcome data. Although these patients might not have needed secondary surgery, to reduce potential bias, we analyzed only those patients who came to follow-up more than 12 months after primary surgery (*n* = 30) in identifying association between obesity status and the presence of secondary surgery. The risk regression analysis ratio was 10.71 (*P* = 0.017).

Common secondary surgeries included tendon transfer/tenodesis and local muscle transfer (67%; 6 of 9). Five patients (56%) underwent a third surgery (Table 3).

**Table 3:** Characteristics of secondary surgery

Patient	Primary surgery	Recovery after primary operation	Indication for secondary surgery	Time between initial injury to secondary surgery (months)	Secondary surgery	Third surgery	Fourth surgery
**1**	Nerve grafting median nerve	No	No recovery of index and thumb flexion	18.3	-**Tenodesis** FDP Ring, little to others		
**2**	Nerve grafting at trunk level	No	No recovery of wrist extension	17.5	-**Tendon transfer** PT to ECRB	Tendon transfer for finger and thumb extension	
**3**	Neurolysis	Partial	Minimal recovery of wrist extension, finger and thumb extension (not function)	24.9	**Tendon transfer** PL to EPL, FCR to ECRB and FCU to EDC	Tenodesis FDP little to others for finger flexion, FDS index tendon transfer fo thumb extension, wrist fusion for finger motion	
**4**	Neurolysis	No	No recovery of elbow flexion	41.8	-**Latissimus dorsi transfer** to distal biceps tendon		
**5**	Neurolysis	Partial	Recovery of shoulder abduction but no recovery of shoulder external rotation	30.8	-**Latissimus dorsi transfer** to infraspinatus insertion	Reattachment Latissimus dorsi insertion (loosening over time)	Reattachment Latissimus dorsi insertion (loosening over time)
**6**	Neurolysis	Partial	Minimal rocovery of shoulder abduction	46.1	**Muscle transfer** to restore shoulder abduction		
**7**	Neurolysis	Partial	Mininal recovery of elbow flexion (not function)	24.8	-Free functional gracilis **muscle transfer** to distal biceps	Latissimus dorsi transfer for elbow flexion	
**8**	Neurolysis	Partial	Minimal rocovery of shoulder abduction	48.3	Shoulder **arthrodesis**		
**9**	Nerve grafting from fifth cervical root to suprascapular nerve and anterior division of upper trunk	Partial	Mininal recovery of shoulder abduction (not function)	28.6	-Transhumeral **amputation**	Shoulder disarticulation	

Time from primary operation to reoperation, median (IQR), m: 12 [9–23]

*FDP* Flexor Digitorum Profundus, *PT* Pronator Teres, *ECRB* Extensor Carpi Radialis Brevis, *PL* Palmaris Longus, *EPL* Extensor Pollicis Longus, *FCR* Flexor Carpi Radialis, *FCU* Flexor Carpi Ulnaris, *EDC* Extensor Digitorum Superficialis, *FDS* Flexor Digitorum Superficialis

## Discussion

The purpose of this study was to determine the association between BMI and the presence of secondary surgery after a poor outcome from primary neurolysis, direct nerve repair or nerve grafting in traumatic brachial plexus injury. We found that obese patients with brachial plexus injury had a 6.99 times higher risk of secondary surgery to improve upper extremity function compared to non-obese patients of the same age and with the same injury severity (high energy injury, associated clavicle fracture, associated shoulder dislocation).

Our study has multiple strengths. First, we used a risk regression model to determine the association between obesity status and the presence of secondary surgery adjusted for age and severity of injury. Second, a manual review of the medical records of identified patients was conducted. We believe that we minimized potential errors from coding. Third, data was obtained over a relatively long period (13 years) from two level 1 trauma centers.

A limitation of this study is that it was not possible control for a confounding factor, time to surgery, in computing the risk ratio due to the inadequate size of the sample.

Our findings are consistent with prior studies which have suggested that size might affect the results of nerve transfer in shoulder reconstruction [17–19, 24], e.g., Socolovsy et al. observed that outcomes of intercostal nerve transfer are better in countries with a lower mean BMI [18]. Other studies have reported that higher BMI is correlated with inferior outcomes after spinal accessory nerve transfer in traumatic brachial plexus palsy [17, 19, 20], but is not correlated with the outcome of elbow flexion restoration [20]. Unlike the present study, the referenced studies analysed the data using correlation, so the effect of BMI was not explicitly demonstrated and there was no adjustment for the effect of potential confounders.

The increased power required to perform motion in the upper extremities might be explained by the higher weight in each part of the upper extremities in obese patients. Additionally, obese status might cause the surgical treatment to be more difficult.

Previously published articles describe an association between age and secondary surgery [25, 26]. Lee et al. found that greater age was associated with poor deltoid recovery after triceps motor branch transfer [17]. Matejcik et al. reported that best results were achieved in patients younger than 20 years [14]. Other studies have also reported that increased age can negatively affect outcomes after nerve repair and nerve grafting in peripheral nerve injury [27–31]. In investigating the association between obesity status and the presence of secondary surgery, we adjusted for age as a confounding factor (Table 2).

The severity of neural injury might affect the outcome of surgery, so we adjusted for severity of injury as a confounder. We further considered that the initial pattern of the injury (C5-C6+/−C7, C8-T1, C5-T1) might not accurately reflect the severity of neural injury. For that reason, in our cohort we used high energy injury, presence of associated clavicle fracture, and presence of associated shoulder dislocation as indicators of the severity of the injury and we controlled for severity of injury as a confounder (Table 2). High energy injury was defined as involving one of the following: motor vehicle accident, severe laceration, and fall from a tree which we suspected that these has high mechanism with wide zone of injury.

The most common secondary surgeries in our study were tendon transfer/ tenodesis and local muscle transfer (67%) followed by free functional muscle transfer (11%), arthrodesis (11%) and amputation (11%) (Table 3). This is consistent with previous experience with brachial plexus surgery. Leffert and Pess reported that 74 brachial plexus injured patients underwent 160 tendon transfers and 94 other additional procedures. More than half of the patients achieved good results [32].

## Conclusions

In obese patients with a brachial plexus injury who undergo neurolysis, direct nerve repair or nerve grafting, the risk of secondary surgery to improve upper extremity function is seven times that of non-obese patients of the same age and with the same severity of injury. A portion of this increased risk may be due to the challenge of powering a heavier upper extremity. A weight reduction program could be considered as the part of the preoperative strategy.

## Supplementary information

**Additional file 1.** List of ICD-9 and CPT codes to identify all patients with traumatic brachial plexus injury who underwent neurolysis, direct nerve repair, or nerve grafting.

## Data Availability

The datasets used and/or analyzed during the current study are available from the corresponding author on reasonable request.

## Abbreviations

MRC: Medical research council
BMI: Body mass index
ICD-9: International classification of disease 9
CPT: Current procedural terminology
IQR: Interquartile range
FDP: Flexor digitorum profundus
PT: Pronator teres
ECRB: Extensor carpi radialis brevis
PL: Palmaris longus
EPL: Extensor pollicis longus
FCR: Flexor carpi radialis
FCU: Flexor carpi ulnaris
EDC: Extensor digitorum superficialis
FDS: Flexor digitorum superficialis
ECRL: Extensor carpi radialis longus
