# Supplementary material for: Obesity status is a risk factor for secondary surgery after neurolysis, direct nerve repair or nerve grafting in traumatic brachial plexus injury: a retrospective cohort study
Source: BMC Surg. 2020 Apr 15;20:73. doi: 10.1186/s12893-020-00737-4 (PMC7160993; doi:10.1186/s12893-020-00737-4)
Supplement: Supplementary file 1 — Additional file 1. List of ICD-9 and CPT codes to identify all patients with traumatic brachial plexus injury who underwent neurolysis, direct nerve repair, or nerve grafting. [file 12893_2020_737_MOESM1_ESM.docx]

**Additional file 1**

ICD-9 codes:

- 353.0: Brachial plexus lesion
- 953.4: Injury to brachial plexus

CPT codes:

- 64713: Under Neuroplasty (Exploration, Neurolysis or Nerve Decompression) Procedures on the Extracranial Nerves, Peripheral Nerves, and Autonomic Nervous System
- 64861: Suture of; brachial plexus
- 64901: Nerve graft, each addition nerve; single strand
- 64897: Nerve graft including harvest, multiple strands, arm, up to 4 cm in length
- 64898: Nerve graft including harvest, multiple strands, arm, greater than 4 cm in length
- 64902: Nerve graft, each addition nerve; multiple strands (cable)
- 64856: Suture of major peripheral nerve, arm or leg, except sciatic; including transposition
- 64857: Suture of major peripheral nerve, arm or leg, except sciatic; without transposition
- 64874: Suture of nerve; requiring extensive mobilization, or transposition of nerve (in addition to nerve suture)
